# Quantitative semiology: harnessing AI-generated teaching signals in psychiatry

**DOI:** 10.1139/jpn-25-0142

**Published:** 2025-10-24

**Authors:** Lena Palaniyappan, Priyadharshini Sabesan

**Affiliations:** ^a^ Douglas Mental Health University Institute, Department of Psychiatry, 6221 McGill University, QC, Canada; ^b^ Department of Psychiatry, Schulich School of Medicine & Dentistry, University of Western Ontario London, ON, Canada; ^c^ Robarts Research Institute & Lawson Health Research Institute, London, ON, Canada

*“Half of us are blind, few of us feel, and we are all deaf.”* – William Osler

## Clinical sense-making in medicine and psychiatry

**Fig. 1.**
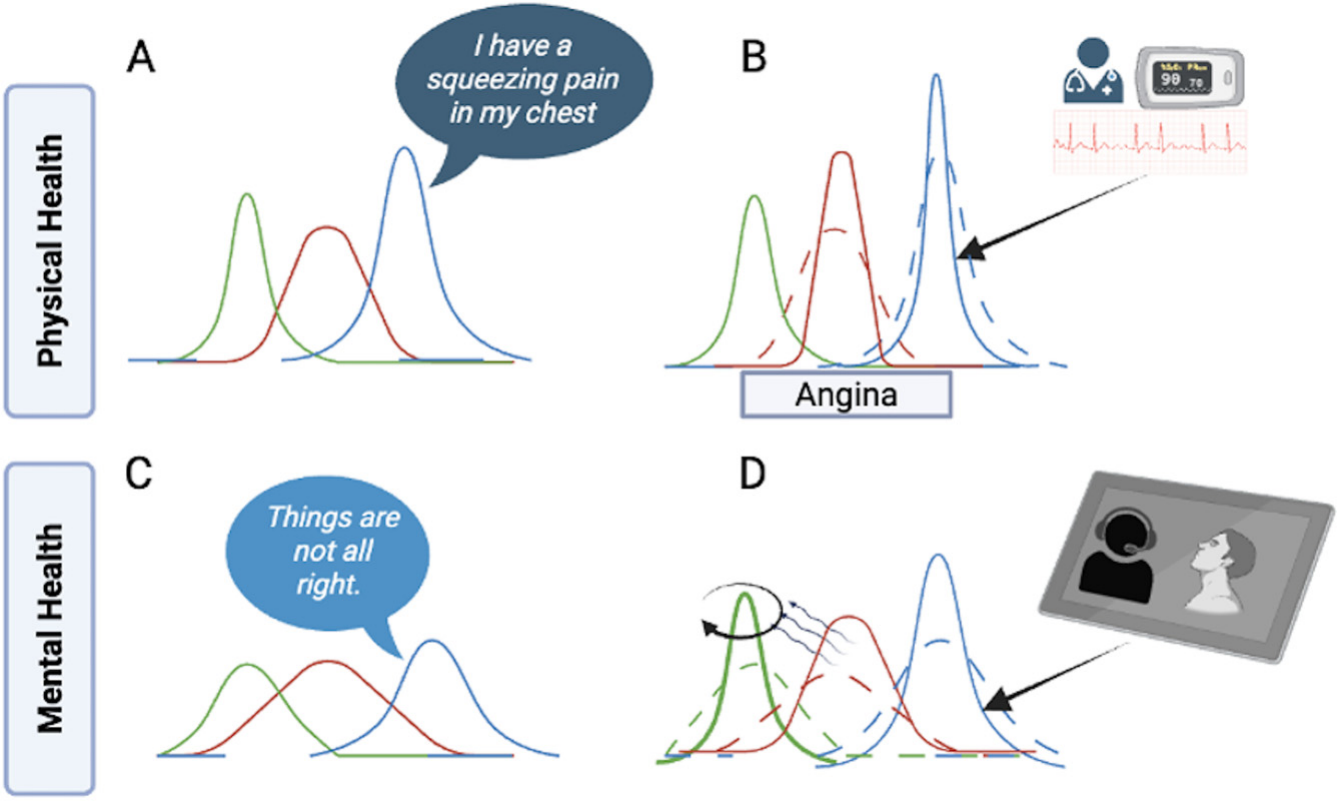
Clinical sense-making as a Bayesian Process. (A) In non-psychiatric medicine, we hold priors about symptoms and diagnosis (green curve), and process patient narratives (blue) against these priors to form inferences that become clinical labels (red). (B) To take certain clinical actions (e.g., thrombolysis or surgery), we require more precise inferences, and we seek further evidence (uninterrupted blue compared to dashed blue) that updates our inferences (change from dashed to uninterrupted red), adding to our clinical confidence. (C) In psychiatric practice, our priors about symptoms and diagnosis are imprecise (green curve), while patient narratives are also fuzzy (blue). The resulting inferences (red) remain imprecise and weak. (D) If the incoming data are more precise (uninterrupted blue compared to dashed blue), mismatches (between dashed and uninterrupted red) will generate teaching signals (wavy lines) that serve to update and refine priors (green curve with a circling arrow). Such precise data can come from focusing on clinical communicative signs and measuring them with audiovisual aids.

A clinician's primary ordeal is deceptively simple: to make sense of another person's suffering and offer solutions. This sense-making, the process of forming a coherent clinical understanding from data presented in various forms, is fundamentally a Bayesian process intuitive to clinicians^1^ (Fig. 1A). We combine prior expectations (prior beliefs) with incoming data to infer the most likely illness labels (posterior beliefs). For example, in a paediatric emergency where seeing a patient with acute abdominal pain is high (prior belief), a child's report of “pain started first around my belly button” (initial incoming data) is likely to invoke an inference of “appendicitis” (posterior belief) but with some uncertainty. This uncertainty is greatly reduced by actively seeking clinical signs (rebound tenderness in right iliac fossa) and ultrasonogram (further evidence). Every mismatch between our expectations and a new wave of incoming data serves to provide a “teaching signal” that updates our expectations and reduces the uncertainty of inferences (Fig. 1B). This process, repeated over time, continuously improves the precision of a clinician's beliefs. In short, teaching signals afford an iterative growth in clinical confidence with every encounter in medicine.^2^

Psychiatry lacks the precision of other medical specialties at multiple levels of this Bayesian sense-making process (Fig. 1C). Many symptoms that we address are in continuum with normal human experience^3^; this means our expectations about target constructs (e.g., symptoms, diagnosis) are often imprecise or fuzzy. At the level of incoming data (i.e., verbatim reports), we receive narratives that are often vague, metaphorical, or fragmented. With fuzzy priors and vague incoming data, the inferences (symptom labels and diagnostic entities) we make remain imprecise and open to criticism.^4^

How, then, do we generate sufficient teaching signals that can improve our clinical sense-making? The JPN has a long tradition of supporting the quest for blood-based or brain-based biomarkers in psychiatry,^5^ which, when realised, could provide new waves of incoming data that aid the clinician. Nevertheless, while we wait for Godot, we can turn to a potential solution hidden in plain sight: clinical signs.

Clinical signs are phenomena that are observable by looking, touching, or listening and can often be measured in physical terms at the proverbial bedside (e.g., the crackles or wheezes in asthma, the degree of abdominal distension in intestinal obstruction). Some obvious examples in psychiatry include the pressured speech in mania, psychomotor slowness in depression, disorganised thinking in schizophrenia, stereotypical repetitions in autism, restless squirming in ADHD, and perseverative behaviors in dementia, to name a few.

## Clinical signs: the missing data stream

When trying to make sense of unclear narratives, we are left with three options: altering our prior beliefs, adding clarity to the vague incoming data (e.g., via rating scales), or looking for additional sources of data (i.e., clinical signs). To understand why signs are our most viable path forward, it is helpful to first consider the alternatives.

The humanistic tradition, led by Karl Jaspers, prescribes a direct manipulation of prior beliefs during clinical sense-making through empathy. Jaspers’ empathetic approach involves “bracketing” one's preconceptions and putting oneself in a patient's shoes to access abnormal mental experiences.^6^^,^^7^ [Perhaps it was Harper Lee's Atticus Finch who articulated this most clearly: “*You never really understand a person until you consider things from his point of view … until you climb into his skin and walk around in it*.”^8^] Requiring a clinician to adopt the patient's perspective, by shedding their own assumptions, is noble in intent, but is difficult to implement consistently in practice. Clinicians often differ substantially from their patients in their cultural and experiential background (see^9^ for more on this topic). Shedding all our preconceptions at will, and changing it 10–12 times in a day, if not impossible, is a real implementational challenge.

The second is the approach of quantitative psychopathology. Proponents of this approach take the view that standardised checklists and rating scales can provide precise measures of the severity of symptoms.^10^ But can we really measure symptoms? Berrios and Markova convincingly argue that symptoms are not natural objects with discernible physical properties for measurement.^11^ Instead, they are “hybrid objects” that emerge when a person becomes aware of a pre-conceptual brain signal and wraps it in a semantic envelope (e.g., “I feel on the edge, as if something bad may happen soon”). The clinician then labels the experience based on prior knowledge (“generalized anxiety”). In this framework,^12^ correct identification of symptoms is contingent on three filters: patient's self-awareness, their choice of verbal descriptors, and the accuracy of clinician's expectations. Any of these filters can affect the clinical inference. This idiosyncratic context-dependency explains why one person's *anxiety* can be another's *depression*. (For another line of argument against quantification of symptoms, see^13^). In sum, quantifying symptom severity using rating scales is like fitting square pegs of ineffable human experiences into our imaginary round holes.

The third alternative, that appears more practical to us, is to complement subjective reports with careful measurement of clinical signs. Unlike symptoms, signs are not subjective experiences reported by patients or filtered by their self-awareness but are observed by clinicians. In contrast from laboratory tests, clinical signs possess ecological validity; that is, they appear not just under fluorescent microscopes but noticeable in ordinary settings, on sidewalks, in living rooms, and at the clinics. Eliciting signs also do not require clinicians to “climb into one's skin and walk around in it”. More importantly, clinical signs offer us the ability to quantify them with physical parameters, e.g., the speed of one's speech and movements, the number of repetitions of a word or a behaviour. This quantifiability yields itself to provide a meaningful index of the severity of the underlying condition. In fact, distinct qualitative phenomena that characterize extremes of quantifiable signs such as motor speed (i.e., catatonic akinesis), speech quantity (i.e., mutism) have been long employed as indicators of prognosis and treatment selection in psychiatry.^14^

Focusing on clinical signs also has other advantages. The severely ill often struggle to describe their experiences as subjective complaints (e.g., catatonia, acute psychotic episode, mania); signs are often the most prominent and reliable features of illness in such situations. Conversely, in very early stages (e.g., prodromal stages), reported symptoms are often non-specific,^15^ reflective of a pluripotent state,^16^ and thus lack precision. Honing in on subtle clinical signs could improve early detection and appropriate intervention.

## Quantifying the observables

As Osler noted (see the opening quote), it is easy to miss clinical signs, especially since patients do not bring them up as problems to be dealt with. This may be why psychiatric practice hardly distinguishes signs from symptoms.^17^ Modern diagnostic manuals have also contributed to the decline in the interest in semiology. For instance, diagnostic signs like odd movements and postures that defined schizophrenia in 16 out of 18 historical criteria are absent from modern criteria.^18^ However, most psychiatric signs are communicative, readily observable without any special maneuvers from our spoken language (verbal) or facial expression, posture, gesture, and tone of voice (non-verbal). Speech technology and the growth of artificial intelligence (AI) now offer unprecedented means to quantify even subtle communicative dysfunctions with sufficient granularity—potentially allaying Osler's concerns on clinicians’ deficiencies. Natural Language Processing (NLP), a branch of applied AI, offers a powerful toolkit to extract and study clinical conversations—capturing *how* something is said, besides *what* is being said.^19^

Speech analysis quantifies speech rate,^20^ rhythm,^21^ and coordination.^22^ Recent studies show NLP can also quantify disorganized speech, a challenging sign often missed by clinicians.^23^ It can be quantified by measuring contextual word probabilities^24^ or the alignment with predictions made by large language models.^25^ Acoustic features and spectrograms can quantify blunted or flat affect,^26^ often overestimated by clinicians.^27^ Some signs that are variations of common communicative phenomena are often underestimated by clinicians. For example, “poverty of content” (speaking much but conveying little information) is a phenomenon that is “readily observable in some of one's colleagues” (McKenna and Oh,^28^ quoting Wing and colleagues^29^) as well as in politicians.^30^ Even such subtle signs can be numerically quantified to track the risk of dementia (idea density)^31^ or schizophrenia (syntactic complexity).^32^

During conversational interactions, automated facial coding can be used to quantify signs related to affect.^33^^,^^34^ As specific facial feature sets represent distinct emotions, we can quantify the rate of change over a period of time, with reduced variance indicating blunting and high variance indicating affective lability.^35–37^ As multi-modal capture and analysis of verbal and non-verbal features becomes more commonplace,^38^ quantifying the mismatch in emotional tone of one's voice and face or between facial features and the context of conversation can provide a readout of affective incongruence. Such automated approaches can extend beyond the face, to capture the body's motion energy to estimate gesture deficits.^39^

In summary, the integration of language science, kinematics, and AI heralds a new era in clinical sense-making. Descriptive psychopathology can now combine subjective and somewhat ephemeral phenomena with features that can be measured, modeled, and monitored. NLP and AI thus offer the tools for quantitative semiology—an empirical approach that transforms subjective observations of signs (e.g., speech pattern, gesture, affect) into objective, quantifiable data using computational tools—a promising route to the next generation of psychopathology.

## Quantitative semiology: more than a measurement

There are several reasons why quantitative semiology of clinical conversations is likely to be more successful than other endeavors towards precision psychiatry. First, more often than not, patients come to us to talk—not to undergo procedures. All our therapies involve talking. Thus, speech is indeed the “biopsy tissue” that is readily available in a psychiatric clinic. Unlike constrained cognitive tests, communicative signs can be measured by recording regular interactions, removing the barrier of motivation to complete a task.

Second, the quantitative semiology approach will allow us to dissociate the act of understanding (can this experience be X?) from that of explaining (why should this experience be X?). AI systems can assist in the latter, potentially freeing clinicians to focus on the former.

Third, in our quest for validating psychiatric constructs, quantitative semiology provides readouts with truly measurable dimensions on which neurobiological measures can be mapped onto. This sets in motion two parallel programs of research—one aiming for neural validation (for example, “does an increase in speech pause length relate to beta oscillatory power in left inferior frontal gyrus?”) and other for practical utility (e.g., “does an increase in pause length predict patient-reported side effect burden of a prescribed medication?”). Several early studies have linked objective speech-based readouts to neural markers. For example, lack of alignment of disorganised speech with predictions made by language models has been linked to glutamate deficiency^40^ and lower excitability of prefrontal cortex^25^ in first-episode psychosis, while increased pausing is seen with the use of antipsychotics with high dopamine (D2) receptor occupancy.^41^

The quantitative semiology of clinical interactions is ideally suited for both in-person and virtual interviews. It is important to note that the measurement of speech, language, and communicative signs in such clinical encounters are conceptually different from, though potentially complementary to, the popular “digital phenotyping” or “mobile sensing” approaches.^42^ For instance, actigraphy measures like steps taken daily, distance moved from home, or smartphone-based counts of number of clicks on an app are all precise physical quantities (quantitative) but not direct signs of one's mental state (i.e., not semiotic in clinical sense). Compare this with the richness of communicative signals that helps many of us to make sense of our spouse's mood. We know what is in store for us from the way that “hello” was spoken on the phone or a video call. Linguistic, acoustic, and facial signals from a conversation provide more face validity when examining someone's mental state. Every word, pause, and inflection carries computationally tractable information about the speaker's mind.

## The future is *less* artificial and more humanistic

Our everyday language is not merely a vehicle for thought; it also constitutes our self and social function. Many psychiatric disorders can be traced back to a dysfunction in the concept of self^43^^,^^44^ with an impact on social function.^45^ Maturana and Varela saw “languaging” as a social coordination process.^46^ Heidegger celebrated language as the “house of being”, emphasizing its role in shaping our concept of self.^47^ Hatab also argues that self is constructed as we “dwell in speech”.^48^ We do not need to look further than our speech, language, and communication to capture the signs of disturbances in self and social function—the two cores of our psychiatric constructs. It is precisely because language is so deeply human that we must approach its quantification with both ambition and caution.

The ambition to harness quantitative semiology from clinical interactions faces formidable challenges that must be addressed to fulfill its therapeutic potential. The reliability of AI models is contingent on the quality and representativeness of their training data; current datasets capturing clinical interactions are rather small.^19^^,^^49^ Even when larger samples are gathered, they are often constrained to homogenous settings (e.g., from randomised clinical trials,^50^ a design that is known to exclude 80% of target population^51^), raising concerns about poor generalizability and transportability to real-world practice. Furthermore, our communicative patterns vary with culture, demographics, and the times we live in; this makes speech markers biosocial rather than biomarkers.^52^ For this reason, we foresee quantitative semiology as primarily a “human-in-the-core” (or “AI-in-the-loop”^53^) approach, rather than an AI-centric approach with a “human-in-the-loop” option^54–56^; the latter are often invoked when building clinical decision systems based on non-semiotic digital markers.

Ultimately, clinical conversations are not just routine exchanges: they are treasure troves of untapped data that blend reflections that are personal and expressions that are social. In an era focused on biomarkers^57^ and symptom checklists,^58^ we risk remaining blind and deaf to the most immediate signals of human suffering: the words, gestures, and voices that meet us every day in the clinic. Quantitative semiology empowers psychiatry to see, hear, and feel with a new kind of acuity: one that is not focussed on granularity but on nuances of human behaviour. The time is ripe for us to actively harness the teaching signals embedded in every clinical conversation. By doing so, we will be standing up to Osler's challenge while we step into AI-assisted psychiatric care.
